# Gadolinium deposition in the brain of dogs after multiple intravenous administrations of linear gadolinium based contrast agents

**DOI:** 10.1371/journal.pone.0227649

**Published:** 2020-02-03

**Authors:** Henning Richter, Patrick Bücker, Calvin Dunker, Uwe Karst, Patrick Robert Kircher

**Affiliations:** 1 Diagnostic Imaging Research Unit (DIRU), Clinic for Diagnostic Imaging, Department of Clinical Diagnostics and Services, Vetsuisse Faculty, University of Zurich, Zurich, Switzerland; 2 Institute of Inorganic and Analytical Chemistry, University of Münster, Münster, Germany; Henry Ford Health System, UNITED STATES

## Abstract

**Objective:**

To determine the effect of a linear gadolinium-based contrast agent (GBCA) on the signal intensity (SI) of the deep cerebellar nuclei (DCN) in a retrospective clinical study on dogs after multiple magnetic resonance (MR) examinations with intravenous injections of gadodiamide and LA-ICP-MS analysis of a canine cerebellum after gadodiamide administration.

**Animals:**

15 client-owned dogs of different breeds and additionally 1 research beagle dog cadaver.

**Procedures:**

In the retrospective study part, 15 dogs who underwent multiple consecutive MR imaging examinations with intravenous injection of linear GBCA gadodiamide were analyzed. SI ratio differences on unenhanced T1-weighted MR images before and after gadodiamide injections was calculated by subtracting SI ratios between DCN and pons of the first examination from the ratio of the last examination. Additionally, 1 research beagle dog cadaver was used for LA-ICP-MS (Laser ablation inductively coupled plasma mass spectrometry) analysis of gadolinium in the cerebellum as an add-on to another animal study. Descriptive and non-parametrical statistical analysis was performed and a p-value of < 0.05 was considered significant.

**Results:**

No statistically significant differences of SI ratios, between DCN and pons, were detectable based on unenhanced T1-weighted MR images. LA-ICP-MS analyses showed between 1.5 to 2.5 μg gadolinium/g tissue in the cerebellum of the examined dog, 35 months after the last of 3 MRI examination with gadodiamide (two examinations at a dose of 1 x 0.1mmol/kg, last examination at a dose of 3 x 0.05mmol/kg).

**Conclusion and clinical relevance:**

Although the retrospective MRI study did not indicate any visible effect of SI increase after multiple gadodiamide exposures, further studies based on LA-ICP-MS showed that the optical threshold was not reached for a potential visible effect. Gadolinium was detectable at a level of 1.5 to 2.5 μg gadolinium/g tissue by using LA-ICP-MS in the cerebellum 35 months after last MRI examination. The general importance of gadolinium retention of subvisible contents requires further investigation.

## Introduction

In 2014 Tomori Kanda[1] published his work about signal intensity (SI) increase on unenhanced T1 weighted magnetic resonance imaging (MRI), causing hyperintensities in the dentate nucleus (DN)-to pons and globus pallidus to thalamus after consecutive injections of gadolinium-based contrast agents (GBCAs). His important work was the beginning of an ongoing debate about the deposition of GBCAs in patient’s brains.[1–18] It is shown in numerous studies on humans and in a few animal studies that there is a positive correlation between SI increase on unenhanced T1 weighted MRI and the gadolinium concentration in the brain.[19–25] Currently, the scientific interest focuses on the question whether all GBCAs or only a specific GBCA subtype is causing such hyperintensities. As free ionic gadolinium is toxic, GBCAs are applied as chelates. According to their chemical structure, two subtypes of GBCAs can be classified. GBCAs of the linear subtype partially and GBCAs of the macrocyclic subtype completely enclose gadolinium. On the one hand, the majority of studies provided evidence that linear GCBAs are stronger correlated with SI increase in the DN.[1, 3–7, 13–15, 17, 18, 20, 22–24, 26–43] On the other hand, SI increase was described after macrocyclic GBCA administration in a few studies that were controversial discussed between specialists due to their limitations.[3, 5, 7, 8, 17] It seems that the molecular structure of the GBCA ligand, which defines the GBCA subtype, is a crucial factor for the SI increase on unenhanced T1 weighted MRI.[44]

In 2017, as a consequence of the scientific debate, regulatory authorities (EMA, European Medicine Agency; FDA Food and Drug Administration) decided about safety issues using GBCAs during clinical work-up, which resulted in divergent actions between EU and USA.[25] Based on a precautionary approach in the European Union nearly all linear GBCAs were removed from the market, while the FDA issued a class warning for all GBCAs.

Until now, the majority of published animal studies about this topic were performed on rodents[23, 24, 45–49] the minority on large experimental animals.[25] So far, in veterinary medicine, no studies described a potential SI increase after multiple linear GBCA administrations in client-owned dogs. This species is of special clinical interest, as dogs will potentially face multiple MR examinations in veterinary medicine during treatment of tumors or during neurological diseases. Moreover, dogs play an indispensable role as animal model for translational research approaches. Therefore, the question arises if in dogs, similar to humans, an SI increase in the DCN is detectable after consecutive MR examinations or whether the number of GBCA administration is below the limit of detection for a visible SI increase on unenhanced T1-weighted MR images. This study followed the guidelines for standardized assessment of SI increase for retrospective MRI studies published from the European Gadolinium Retention Evaluation Consortium (GREC).[44]

In the current study, we aimed to retrospectively assess clinical data of dogs after multiple MRI examinations with intravenous administration of gadodiamide. Additionally, the brain of one dog, which was euthanized unrelated to this study, underwent an LA-ICP-MS measurement of gadodiamide 35 months after last MRI examination.[50–52] We hypothesized that there is an increased hyperintensity on non-enhanced T1 weighted sequences in the deep cerebellar nucleus (DCN) of dogs after multiple gadodiamide administrations and a detectable gadodiamide retention in the cerebellum of dogs with LA-ICP-MS.

## Material and methods

### Dataset of patients

This restrospective study was performed on MR imaging data sets of 18 client-owned dogs presented between August 2012 and October 2017 at the Clinic for Diagnostic Imaging at the Vetsuisse Faculty of the University of Zurich. Inclusion criteria was that the dogs had multiple (defined as two or more) MR examinations of the brain with intravenous administration of the linear GBCA gadodiamide at a dose of 0.15mmol/kg. Three patients did not undergo unenhanced T1-weighted imaging or were unreadable for study purposes and were excluded from this study. However, 15 client-owned dogs met the inclusion criteria and had a history of neoplastic or neurological disease as reason for MRI examination. Demographic and clinical characteristics of the all animals in the dataset is summarized in Table 1. All animals underwent general anesthesia during MRI examination based on a standard anesthesia protocol. After premedication with butorphanol and continious intravenous lactated Ringer’s infusion, general anesthesia was induced with propofol and maintained with isoflurane combined in oxygen and air. One research beagle dog cadaver, which was euthanized unrelated to this study, was used for dissection and sampling of the whole brain. Accordingly, the additional organ sampling, which was an add-on to the main study purpose, applied 3R requirements and maximized the scientific output from the dog. LA-ICP-MS was used for determination of Gd-concentration in the cerebellum.

**Table 1:** Demographic and clinical characteristics.

animal	BW [kg]	number of GBCA applications	cumulated dose [mmol/animal]	time between last GBCA and last MRI [days]	gender	breed	type of disease
1	11.2	2	3.36	254	female, neutered	Spitz	meningioma
2	33.0	2	9.90	169	male	Labrador Retriever	meningioma
3	37.0	3	16.65	98	male	Boxer	unspecified recurrent brain tumor
4	15.5	2	4.65	77	male, neutered	franz. Bulldogge	pituitary tumor
5	24.6	2	7.38	92	female, neutered	Magyar Vizsla	meningioma
6	6.0	2	1.80	137	female, neutered	Jack Russel Terrier	meningioma
7	13.2	2	3.96	582	male, neutered	franz. Bulldogge	cushing
8	1.8	2	0.54	138	male	Yorkshire Terrier	meningoencephalitis
9	22.8	2	6.84	83	female, neutered	Katalanischer Schäferhund	meningoencephalitis
10	29.0	2	8.70	24	male	Boxer	unspecified recurrent brain tumor
11	29.5	2	8.85	114	male, neutered	Collie	meningioma
12	22.7	2	6.81	374	male, neutered	Labrador Retriever	meningioma
13	35.5	2	10.65	135	male	Boxer	unspecified recurrent brain tumor
14	8.5	2	2.55	217	male, neutered	Jack Russel Terrier	pituitary tumor
15	22.4	3	10.08	106	female, neutered	Labrador Retriever	pituitary tumor
16	11.4	3	5.13	552	female	beagle	healthy
**median**	**22.6**	**2**	**6.83**	**136**			
**min**	**1.8**	**2**	**0.54**	**24**			
**max**	**37.0**	**3**	**16.65**	**582**			

Demographic and clinical characteristics of all dogs displayed with date of birth (DOB), age at last MRI [months], bodyweight (BW) [kg], number of GBCA applications, cumulated dose [mmol/animal], time between last GBCA and last MRI [days], gender, breed, and type of disease.

### Imaging and data analysis

The analysis included whole-brain MR imaging obtained between 2016 and 2019 at the Vetsuisse Faculty Zurich. Imaging was performed with a 3.0-T MRI (Philips Ingenia, Philips Netherlands). Transverse unenhanced T1-weighted image parameters were as follows: Repetition time: 11.15–13.17 ms, Echo time: 5.116–6.125 ms, slice thickness: 0.7 mm, number of averages: 1, acquisition matrix: 0/228/227/0 and echo train length: 227.

**Fig 1:**
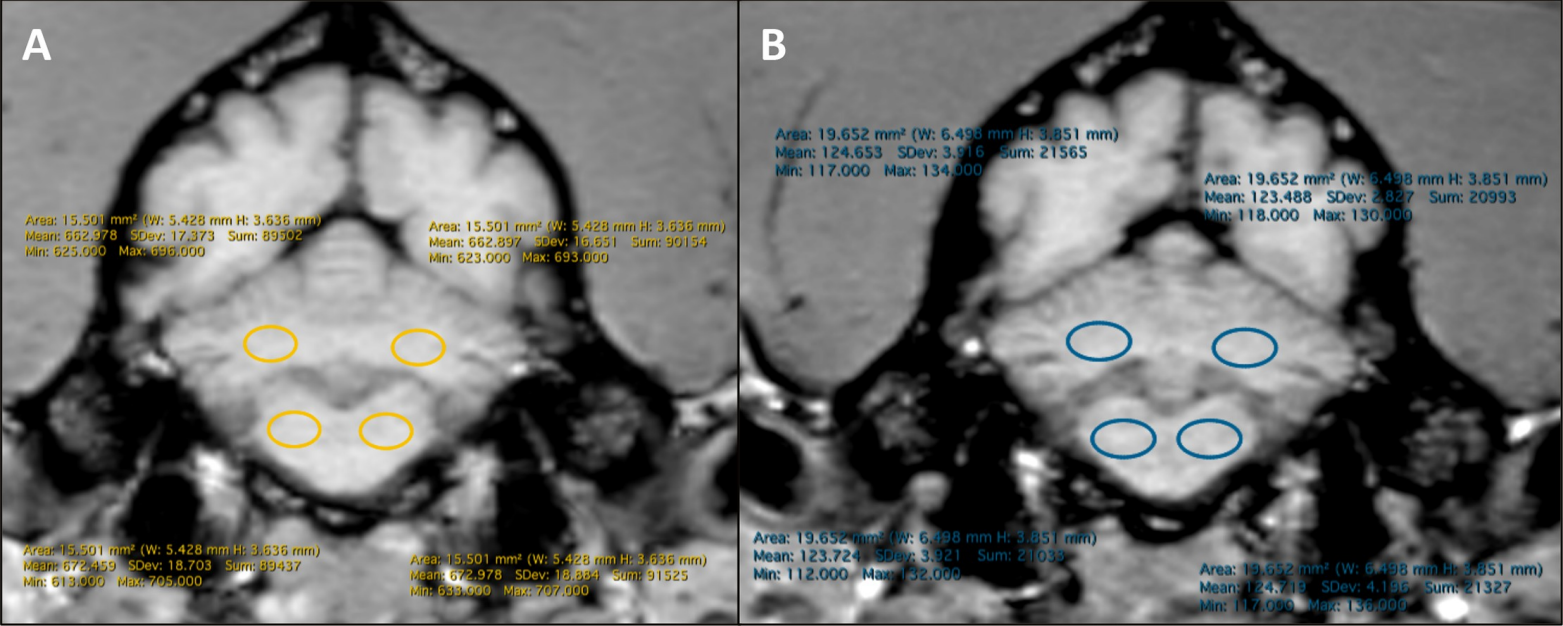
T1 weighted unenhanced images a dogs brain in transversal orientation at the level of DCN; Colored circles indicate ROI at the Pons (proximal circles) and DCN (distal circles) in the left and right hemisphere **A:** first examination precontrast, **B:** second examination precontrast 105 days after first examination; related SI measurements of the ROIs showing measured area in mm^2^, Mean, Min, Max and SD of SI in the ROI.

Image analysis was performed independently by two of the authors (HR, PK). A picture archiving and communication system (Synapse^®^ PACS, Fujifilm) was used for all reading sessions. Evaluation of the images, including analysis of ROIs and the mean SI values, was performed with open source medical image viewer (Horos based upon OsiriX^TM^, 64-bit medical image viewer for OS X, Version 3 (LGPL-3.0)). Pre- and postcontrast images were subjectively compared and analyzed regarding presence of signal alteration in the region of the deep cerebellar nuclei (DCN). Regions of interest (ROI) were drawn on the unenhanced T1-weighted images on the central pons, and the DCN on both hemispheres. (Fig 1) The anatomically correct description of the DN in dogs is *Nucleus lateralis cerebelli*, which is the most prominent DCN. Accordingly, we use the more generalized term DCN instead of DN or *Nucleus lateralis cerebelli* to better reflect the canine anatomical situation. The correct placement of the ROI was confirmed by using T2-weighted images at the same section position for identification of the DCN in unclear cases. The DCN-to-pons SI ratio was calculated by using the following formula:

DCN‐to‐ponsSIratio=(meanSIofDCN)/(meanSIofcentralpons).

The first and last MR examination of the dogs was used to calculate the SI ratio difference by substracting the first examination SI ratio from the last examination SI ratio.

### Cadaver sample preparation

The research beagle dog was euthananized according to another unrelated study (animal license number ZH057/17) in accordance to Swiss animal welfare act. As an add-on to the main study, the dog's brain was harvested directly after euthanasia. The left brain hemisphere was coronally cut into 0.5–1.0 cm slices and cryopreserved (at ^−^80°C). Deep cerebellar nuclei of the left cerebellum were cut into a 50-μm-thick section and fixed on a piece of cork with Tissue-Tek O.C.T. Compound (Sakura Finetek GmbH, Staufen, Germany). For chemical analysis, the deep cerebellar nuclei were cut in thin sections of 10-μm thickness with a cryotome and mounted on microscopic glass slides. Before the ablation process, microscopic images were recorded with a BZ-9000 inverted fluorescence/bright field microscope (Keyence, Osaka, Japan). The right hemisphere of the brain was formalin fixed (4% buffered formalin), paraffin embedded, and stored until further analysis.

### Localisation of deep cerebral nuclei by bench-top μXRF

For the localisation of deep cerebral nuclei, an M4 Tornado bench-top μXRFinstrument (Bruker Nano GmbH, Berlin, Germany) was used. The rhodium X-ray tube was supplied with a voltage of 50 kV and a current of 600 μA throughout the measurements. The emitted X-ray fluorescence was detected by a silicon drift detector (XFlash® 5030, Bruker Nano GmbH, Berlin, Germany). Spatial resolution was approximately 25 μm. Each pixel was measured twice for 200 ms at a pressure of 20 mbar. The data was evaluated using the software ESPRIT HyperMap (Bruker Nano GmbH, Berlin, Germany).

### Standard preparation for LA-ICP-MS

For calibration, 11 matrix-matched gelatine standards in a concentration range from 0 ng/g to 600 μg/g were created. For this purpose, a 1000 μg/g gadolinium (Gd) ICP-MS standard (Fluka Analytical, St. Gallen, Switzerland) was diluted and combined with gelatine of highest purity (Grüssing GmbH, Filsum, Germany) resulting in standards with a gelatine content of 10% (w/w). The suspension was homogenized by heating the standards to a temperature of 50°C and the usage of a vortex mixer. Afterwards, 10 μm thin slices of each standard were prepared via a CryoStar NX70 Cryostat (Thermo Fisher Scientific, Waltham, USA). The thin slices of the standards where then mounted onto a microscopic slide.

The Gd concentration in the gelatine standards was confirmed by ICP-TQMS (iCAP TQ, Thermo Fisher Scientific, Waltham, USA) analysis of the digested standards. For digestion, 500 μL concentrated nitric acid (Merck Chemicals GmbH, Darmstadt, Germany) and 100 μL 35% (w/w) hydrogen peroxide (Acros Organics, Geel, Belgium) were added to around 50 mg of each gelatine standard. The mixture was then heated to a temperature of 70°C until complete digestion. Afterwards, the digests were diluted and a rhodium (Rh) ICP-MS standard (SCP Science, Baie-D’Urfe, Canada) was added as an internal standard. The concentration was determined using an external Gd calibration in a range from 0 pg/g to 30 ng/g consisting of 12 standards. Again, Rh was used as an internal standard.

### LA-ICP-MS measurements

For elemental mapping of the Gd distribution, the hyphenation of an LSX 213 G2+ (Cetac Technologies, Omaha, USA) laser ablation system equipped with a HelEx II cell (Teledyne Cetac Technologies, Omaha USA) and an ICP-MS 2030 (Shimadzu, Kyoto, Japan) was used. For ablation, a spot size of 10 μm in combination with a stage speed of 30 μm/s was chosen. Laser energy was optimized for each individual sample to allow for quantitative ablation of the sample with minor ablation of the object slide. Ablated particles were washed out by a constant helium flow of 800 mL/min. For more efficient transportation, a daily tuned argon flow was added on the line to the micro torch of the ICP-MS system. A wet argon plasma with an RF power of 1200 W was used for the ionisation. The ionized analytes were led through a nickel interface and were analysed by an SQ-KED setup.

For quantification, the previously described matrix-matched Gd gelatine standards were used. On each standard, 11 lines with an ablation time of 20 s per line were ablated with the same parameters as used for the sample. The first line of each recording was discarded for consistency reasons.

All data evaluation was performed using the software ImaJar (developed by Robin Schmid, Muenster, Germany).

### Statistics

Statistical data was analyzed by using SPSS (IBM^®^ SPSS^®^ Statistics, version 25, 64-bit-version, IBM, Chicago, Ill). Due to the limited number of available cases, data was defined as non-normally distributed and quantitative data was presented with median (range). Non-parametrical tests were used to compare SI ratio differences between 2 independent observers (Mann-Whitney U test). Inter-rater reliability was assessed based on Intraclass-Correlation-Coefficient (ICC) in a range from 0.0 to 1.0, whereby large numbers mean better reliability. One-sample t-tests were used to examine if the mean SI ratio differences between first and last examination were different from 0. A p-value of < 0.05 was considered significant.

## Results

### Cinical and demographic aspects

An overview about the demographic and cinical characteristic of the dataset is available provided as Table 1. The mean age of all dog patients included was 96.20 (31–157) months. The animals gender was divided as followed: male (5), male castrated (5), female castrated (5). As this was a restrospective clinical study, the dogs were of different breeds: Boxer (3), Border Collie (1), French Bulldog (2), Jack Russel Terrier (2), Catalan Scheepdog (1), Labrador Retriever (3), Magyar Vizsna (1), Spitz (1), Yorkshire Terrier (1). Contrast media injection protocols were the same for all animals, as they followed a clinical standard operation procedure with 0.15 mmol/kg gadodiamide for each MRI examination. The time between the MRI examinations was caused by the clinical work-up and was in median 136 (24–582) days.

Additionally, one research beagle dog cadaver was included into this study, which was euthanized for another unrelated study. The research beagle dog was used for different studies including repetitive gadodiamide administrations in MRI before his termination. It was a 52 months old, female castrated beagle dog with 11.4 kg body weight. The contrast media injections of the dog were as followed: November 2013 (0.1 mmol/kg gadodiamide), December 2013 (0.1 mmol/kg gadodiamide), June 2015 (3 x 0.05 mmol/kg gadodiamide). Accordingly, the last gadodiamide injection was 35 months before termination. Following the 3R requirements, the brain of the dog was sampled and used for chemical analysis, as this was not possible to perform with the retrospectively examined clinical patient population.

### MRI data

Unenhanced T1-weighted imaging were independenly analyzed by two observers (HR, PK). A summary of all measurements are provided as Table 2. SI values of the DCN and Pons as well as the SI ratios and the SI ratio differences between the first and the last examination of the left and right hemisphere are summarized in Table 3. Based on non-parametric Mann-Whitney U tests, no significant differences were detectable between both observers at a significance level of p< 0.05 (SI DCN left (p = 0.988), SI Pons left (p = 0.882), SI ratio left (p = 0.329), SI DCN right (p = 0.976), SI Pons right (p = 0.894), Si ratio right (p = 0.605), SI ratio difference left (p = 0.756), SI ratio difference right (p = 0.548).

**Table 2:** SI ratio differences.

	observer 1								oberver 2							
animal	SI DN left	SI Pons left	SI ratio left	SI DN right	SI Pons right	SI ratio right	SI ratio difference left	SI ratio difference right	SI DN left	SI Pons left	SI ratio left	SI DN right	SI Pons right	SI ratio right	SI ratio difference left	SI ratio difference right
1	125.053	126.691	0.987	124.613	125.208	0.995			124.606	126.761	0.983	125.667	125.343	1.003		
1	277.428	281.183	0.987	280.295	287.391	0.975	0.000	-0.020	272.885	282.305	0.967	279.496	285.953	0.977	-0.016	-0.025
2	366.1	372.94	0.982	355.652	361.514	0.984			471.703	468.736	1.006	460.095	466.525	0.986		
2	362.487	359.643	1.008	360.836	355.127	1.016	0.026	0.032	362.179	360.978	1.003	357.583	361.620	0.989	-0.003	0.003
3	172.257	173.655	0.992	174.972	176.618	0.991			173.935	176.551	0.985	176.775	177.906	0.994		
3	178.492	183.944	0.970	179.350	184.430	0.972	-0.022	-0.018	179.023	183.55	0.975	175.918	186.732	0.942	-0.010	-0.052
3	422.66	449.613	0.940	437.129	459.989	0.950	-0.052	-0.040	419.148	455.787	0.920	429.200	458.764	0.936	-0.066	-0.058
4	175.919	172.502	1.020	172.275	175.804	0.980			172.698	175.878	0.982	173.125	175.544	0.986		
4	343.066	340.951	1.006	344.929	346.556	0.995	-0.014	0.015	349.659	342.713	1.020	350.028	348.415	1.005	0.038	0.018
5	614.737	628.453	0.978	639.614	647.677	0.988			622.014	632.662	0.983	634.766	652.891	0.972		
5	643.488	642.776	1.001	647.769	635.415	1.019	0.023	0.032	648.4	653.633	0.992	650.581	640.549	1.016	0.009	0.043
6	423.872	427.325	0.992	430.475	429.126	1.003			430.852	439.226	0.981	431.025	431.684	0.998		
6	1337.617	1308.215	1.022	1350.109	1308.886	1.031	0.031	0.028	1345.156	1341.79	1.003	1347.488	1345.814	1.001	0.022	0.003
7	997.558	993.47	1.004	995.635	1003.037	0.993			988.921	1000.341	0.989	998.839	1004.823	0.994		
7	590.895	589.339	1.003	580.894	577.059	1.007	-0.001	0.014	581.192	591.546	0.982	572.667	593.458	0.965	-0.006	-0.029
8	121.255	124.544	0.974	122.963	116.500	1.055			122.184	124.16	0.984	122.953	117.417	1.047		
8	149.632	134.637	1.111	129.449	129.388	1.000	0.138	-0.055	148.141	138.991	1.066	126.260	128.084	0.986	0.082	-0.061
9	97.817	88.727	1.102	95.320	90.399	1.054			98.402	90.347	1.089	95.379	90.695	1.052		
9	97.977	95.76	1.023	97.402	95.758	1.017	-0.079	-0.037	97.07	95.184	1.020	97.455	95.400	1.022	-0.069	-0.030
10	131.948	133.301	0.990	130.471	136.535	0.956			128	134.089	0.955	130.690	137.105	0.953		
10	120.822	125.631	0.962	121.811	123.667	0.985	-0.028	0.029	112.01	122.136	0.917	110.887	122.901	0.902	-0.037	-0.051
11	133.753	134.268	0.996	134.603	134.671	0.999			134.295	133.571	1.005	136.521	136.333	1.001		
11	129.713	124.613	1.041	129.773	121.503	1.068	0.045	0.069	130.938	126.121	1.038	127.061	122.018	1.041	0.033	0.040
12	76.143	78.517	0.970	77.633	78.761	0.986			73.75	78.213	0.943	76.652	79.258	0.967		
12	86.485	95.423	0.906	85.735	99.646	0.860	-0.063	-0.125	84.317	95.434	0.884	85.253	96.134	0.887	-0.059	-0.080
13	707.581	726.456	0.974	690.724	692.353	0.998			713.182	735.14	0.970	695.767	704.060	0.988		
13	626.935	637.184	0.984	611.346	622.576	0.982	0.010	-0.016	615.634	624.683	0.986	629.760	611.041	1.031	0.015	0.042
14	504.183	517.667	0.974	514.801	519.431	0.991			509.696	522.529	0.975	517.467	513.294	1.008		
14	136.327	138.181	0.987	137.012	139.957	0.979	0.013	-0.012	136.547	138.476	0.986	136.415	138.630	0.984	0.011	-0.024
15	101.85	96.083	1.060	101.685	94.867	1.072			103.259	96.485	1.070	103.662	96.792	1.071		
15	123.488	124.719	0.990	124.653	123.724	1.008	-0.070	-0.064	125.277	123.168	1.017	125.057	127.250	0.983	-0.053	-0.088
15	662.897	672.978	0.985	662.978	672.459	0.986	-0.075	-0.086	659.586	677.183	0.974	660.568	680.373	0.971	-0.096	-0.100
**median**	** **	** **	**0.990**	** **	** **	**0.994**	**-0.001**	**-0.016**	** **	** **	**0.985**	** **	** **	**0.989**	**-0.006**	**-0.029**
**min**	** **		**0.906**			**0.860**	**-0.079**	**-0.125**	** **		**0.884**			**0.887**	**-0.096**	**-0.100**
**max**	** **	** **	**1.111**	** **	** **	**1.072**	**0.138**	**0.069**	** **	** **	**1.089**	** **	** **	**1.071**	**0.082**	**0.043**

SI ratio differences from the two observers for each single dog, as well as median, min and max of each measurement

**Table 3:** Measurement data.

		observer 1					observer 2					Mann-Whitney U test
measurement	N	minimum	maximum	mean	standard deviation	One-sample T-test	minimum	maximum	mean	standard deviation	One-sample T-test	p-value
SI DCN left	30	76	1338	358	307		74	1345	361	308		0.988
SI Pons left	30	79	1308	360	306		78	1342	366	311		0.882
SI ratio left	30	0.906	1.111	0.999	0.041	0.862	0.884	1.089	0.989	0.043	0.158	0.329
SI DCN right	30	78	1350	358	308		77	1347	361	310		0.976
SI Pons right	30	79	1309	359	305		79	1346	365	311		0.894
SI ratio right	30	0.860	1.072	0.997	0.039	0.701	0.887	1.071	0.991	0.040	0.227	0.605
SI ratio difference left	15	-0.079	0.138	-0.002	0.055	0.896	-0.096	0.082	-0.010	0.048	0.451	0.756
SI ratio difference right	15	-0.125	0.069	-0.011	0.051	0.402	-0.100	0.043	-0.021	0.045	0.098	0.548

Measurements of observer 1 and 2 displayed as mean, min, max and SD for DCN and Pons, SI ratio and SI ratio difference in the left and right hemisphere. One-sample t-test for both hemispheres, testing for differences from 1 (for SI ratios), respective from 0 (SI ratio differences). Additional p- values of the Mann-Whitney-U test comparing results of oberserver 1 and 2.

The ICC showed very good agreement between both observers (Table 3). For all analyses, ICC was between 0.863 (SI ratio difference right hemisphere)– 0.999 (SI DCN left, SI Pons left, SI DCN right, SI Pons right).

SI ratio DCN-to-pons showed no significant differences between left and right hemisphere (observer 1 (p = 0.390), observer 2 (p = 0.722)). SI ratio DCN-to-pons at the right hemisphere was measured in median with 0.997 (0.860–1.072) for observer 1 and 0.991 (0.887–1.071) for observer 2, and at the left hemisphere with 0.999 (0.906–1.111)) for observer 1 and 0.989 (0.884–1.089) for observer 2. (Table 4)

**Table 4:** Intraclass-correlation coefficient (ICC).

		ICC	confidence interval	
measurement	hemisphere	mean	min	max
SI DCN	left	0.999	0.998	0.999
SI Pons		0.999	0.998	1.000
SI ratio		0.994	0.989	0.999
SI ratio difference		0.949	0.847	0.983
SI DCN	right	0.999	0.998	1.000
SI Pons		0.999	0.998	0.999
SI ratio		0.910	0.811	0.957
SI ratio difference		0.863	0.592	0.954

Intraclass-correlation coefficient (ICC) between observer 1 and 2. Displayed as mean and confidence interval for SI measurements at the DCN and Pons, for SI ratios and SI ratio differences in the left and right hemisphere

SI ratio differences between the first and the last MRI examination of the patients showed no significant differences, whether at the left nor at the right hemisphere (observer 1 (p = 0.831), observer 2 (p = 0.163)). SI ratio differences between the first and the last MRI examination of the patients was measured at the right hemisphere in median with -0.011 (-0.125–0.069) for observer 1 and -0.021 (-0.100–0.043) for observer 2, and at the left hemisphere with -0.002 (-0.079–0.138) for observer 1 and -0.010 (-0.096–0.082) for observer 2. (Table 1) Based on a one-sample t-test, the first and the last MRI examination was tested to be significant different from 0. For both observers, no significant difference from 0 was found (observer 1: p = 0.896/0.402; observer 2: p = 0.451/0.098 [left/right]).

### LA-ICP-MS data

**Fig 2:**
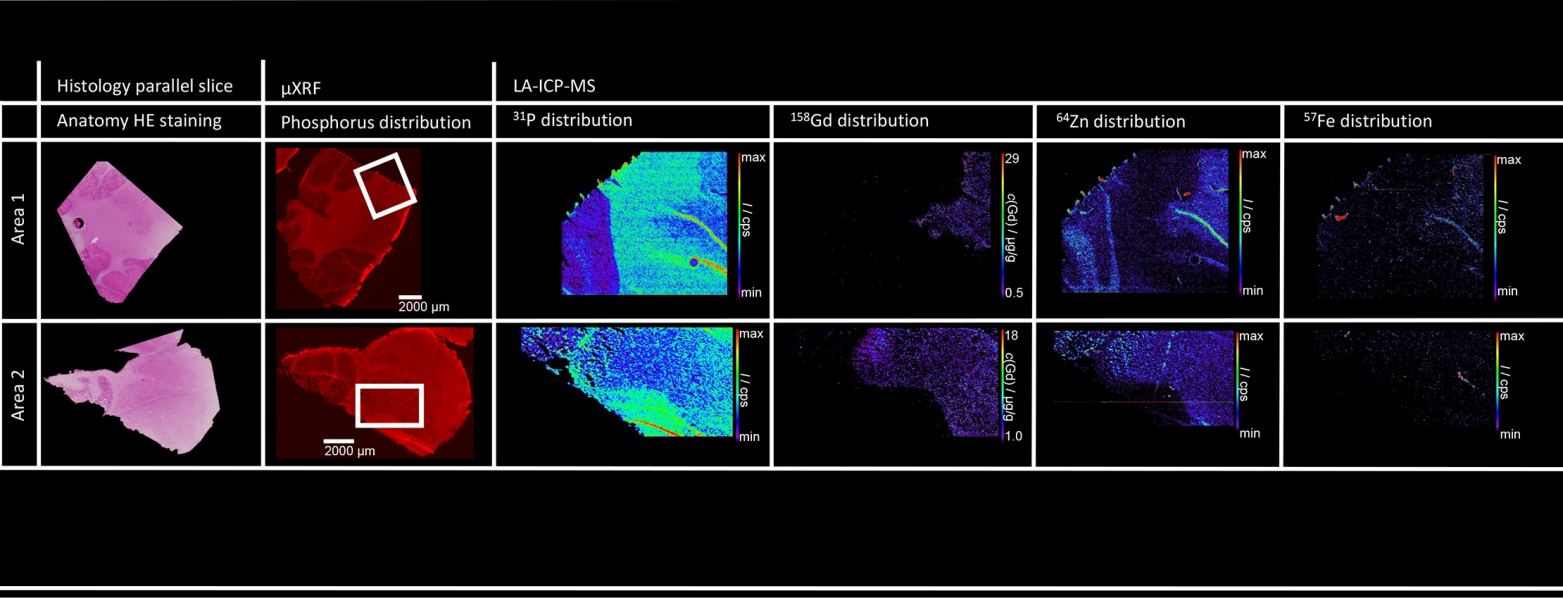
Representative results for the LA-ICP-MS analyses of two cryo cerebellum samples of one dog treated with gadodiamide 35 months before euthanasia. Anatomy is shown on histological and μXRF images. LA-ICP-MS results are displayed as quantitative distribution map of gadolinium (^158^Gd) and as qualitative distribution maps of phosphorus (^31^P), iron (^57^Fe) and zinc (^66^Zn). LA-ICP-MS analyses were performed with a laser spot size of 10 μm and a limit of detection at 67 ng/g (LOD). A clear colocalization of gadolinium and zinc displays in both samples.

Visual assessment of LA-ICP-MS analysis as well as a quantitative analysis was performed with a limit of quantification (LOQ) of 220 ng gadolinium/g tissue and a limit of detection (LOD) of 67 ng/g. Gadolinium concentrations in the DCN of the research beagle dog could be determined between 1.5 to 2.5 μg gadolinium/g tissue, 35 months after last gadodiamide injection. In agreement with the visual assessment of the DCN, gadolinium levels were higher in the DCN than in the surrounding area of the DCN. A co-localisation of Gd and zinc (Zn) was detectable, both increasingly detectable in vessels, especially at the DCN. Furthermore, an anti-correlation with phosphorus (P) was detectable, which is visible in μXRF and LA-ICP-MS. The results are summarized in Fig 2.

## Discussion

The current restrospective clinical study is the first study observing multiple gadodiamide applications in veterinary patients in a clinical set-up. The background of this study was the ongoing debate about the clinical relevance of hyperintensities of the DCN after multiple GBCA applications in humans. The hypothesis of this study was that there is a detectable increased hyperintensity on non-enhanced T1 weighted sequences in the DCN of dogs after multiple gadodiamide administrations and a measurable gadolinium retention in the cerebellum of dogs after chemical analysis based on LA-ICP-MS. The hypothesis has to be partially rejected.

This study showed 1) no hyperintensities on non-enhanced T1 weighted sequences in the clinical dog patients and 2) a Gd concentration of 1.5 to 2.5 μg gadolinium/g tissue in the brain of a research beagle dog 35 months after last gadodiamide injection.

With these findings, our study supports the hypothesis that a specific threshold is needed to identify hyperintensities on unenhanced T1-weighted images. It seems likely that the data of this dog patient population was not exposed to gadodiamide in an amount to exceed this threshold. With a median cumulative dose of 6.83 mmol/animal (range 0.54 to 16.65mmol/animal) the clinical dataset shows much less cumulative GBCA dose as compared to published reports from human medicine [1] or animal experiments.[53] Even if a superior brain clearance of macrocyclic GBCAs over linear GBCAs is described[54, 55] and all dogs of this study got a linear GBCA administration, the cumulative dose did not reach a visible level of hyperintensities after 2–3 administrations. We determined mean SI ratio differences between first and last MRI examination between 0.2% and 6.9% (range -12.5% to 13.8%), which was not significantly different from 0. (Table 1) This is much lower than in the study of Hu et al., were an SI increase between first and most recent MRI examination with an increase of 18.6% ± 12.7% (range 0.5% to 47.5%) for the DCN was reported.[14] In the study of Hu et al., human patients received a significant number of subsequent GBCA examinations, ranging between 5 and 37. Weberling et al. described a significant SI increase in the DCN after at least 5 consecutive GBCA injections of gadobenate dimeglumine.[13]

Although the retrospective MRI study did not indicate any visible effect of SI increase after multiple gadodiamide exposures, further studies based on LA-ICP-MS showed that the optical threshold was not reached for a potential visible effect. Most of the dog patients in this study had a history of neoplastic or neurological disease as reason for MRI examination (Table 1). In the patient records of the dogs, no side effects were linked with the contrast media application. This result is of clinical relevance for all current and future patients in veterinary medicine, which have a history of diagnosed brain tumors (such as meningiomas gliomas, nerve sheeth tumors, pituitary adenomas) or meningoencephalitis. Recurrent MRI examination are of interest in veterinary medicine mainly in dog patients after radiation therapy for follow ups and/or recidive pathologies. Our dataset showed that mostly not more than two MRI examinations with GBCA are performed during clinical work-up in veterinary medicine. This is a clear difference in comparison with human medicine, were more than 35 linear GBCA administrations to a single patient are reported.[18]

Even if there is no clinically visible hyperintensity on unenhanced T1-weighted MR images, this does not mean there is no deposition of gadolinium through gadodiamide application after multiple contrast media administrations in dogs. From human medicine it is known that approximately 6 injections of linear GBCAs are needed, before hyperintensities in the DCN become detectable on MRI[7, 56] On the other hand, gadolinium deposition was detectable after a single injection of linear GBCAs based on LA-ICP-MS measurments in the brain of sheep.[25] This study confirms gadolinium detection in the brain of a research beagle dog based on the chemical analysis of the Gd content. After three examinations, two times with 1x0.1 mmol/kg and once with 3x0.05 mmol/kg gadodiamide, Gd was measurable 35 month after the last administration. Accordingly, it could be shown in this study that the described Gd deposition in the brain of different species similarly occurs in dogs. Therefore, our findings support the hypothesis of irreversible or long lasting Gd deposition at the DCN. The current explanation about the pathomechanism is based on de-chelation effects of linear GBCA and transmetalation in co-localization with other elements, such as Fe, Co, Cu, Zn.[25, 57] We could show similar effects in the research beagle dog, which supports the ongoing discussion. As dogs are a widely used species for translational medicine and model for lots of pre-clinical studies during the approval of new medical products for human medicine, the results of our study should be recognized for all translational studies in future.

Even if the pathomechanism is not clarified at the moment and there are ongoing studies about differences between linear and macrocyclic GBCAs, from a scientific and ethical point of view it is mandatory to reduce the GBCA administration to the essential number with diagnostic importance for the patients. At the same time it is important to maintain the benefit of GBCA as long as there are no valuable alternative contrast media available. This should be done with respect to the risk of Gd deposition and its accumulation during recurrent contrast agent administrations.

As one of the limitations of this study, we have to mention that the number of patients and the number of gadodiamide administrations was lower than in comparable restrospective human studies. The reason is that in veterinary medicine dogs, as the most used private owned species, will not be imaged more often in MRI. This can be explained by the costs (payed by the owner) and the shorter life-span of a dog in comparison to humans. As our data reflects the daily business in a large university hospital, this was the maximum available data, which provides a relevant veterinary insight into this topic. Unfortunately, we did not have an MRI of the research beagle dogs brain, who had multiple gadodiamide administrations before euthanasia. This was caused by the fact that the brain of the dog was used as an add-on to another unrelated study which focused on other regions than the animals brain during MRI examination. It is a beneficial circumstance to have the results of the chemical analysis from this dog, even if there is no associated MRI of the brain. Based on the gained knowledge that an increased signal intensity of the brain might only be visible when the number of GBCA injections surpasses a certain threshold[34], it may have been questionable if this threshold would have been exceeded or not. According to the current knowledge, gadolinium first becomes visible in MRI if a threshold of approximately 1 μg gadolinium/g tissue is exceeded.[25] We assessed between 1.5 to 2.5 μg gadolinium/g tissue in the DCN of the research beagle dog, which would have caused a signal intensity change in MRI. Currently published case reports and research studies describe LA-ICP-MS as a useful tool to measure the quantities of retained gadolinium in human[19, 58, 59] and rodent tissues[47, 60]. Potential risk factors with effect on Gd retention described are ongoing neuroinflammation[60] or primary gliomas[59]. Additionally, any type of inflammation can negatively influence the blood brain barrier permeability[61] and therefore could be potential risk factor for gadolinium retention. In this study LA-ICP-MS was performed in the brain of a healthy research beagle dog, which was considered to be not influenced by clinical factors. On the other side the dataset of this study includes patients showing exactly such types of diseases with potential effect on gadolinium retention. The question how and in which quantity the underlying diseases or a reduced blood-brain-barrier influenced the gadolinium retention in the brain can not be estimated and has to be evaluated in further studies.

Another potential confounding factor for all clinical studies is the time between last GBCA administration and the MRI examination to detect hyperintensities in the DCN or until LA-ICP-MS measurement of gadolinium, as this time is crucial to differentiate between the soluble and insoluble form of gadolinium. In this study the median number of days between last GBCA application and MRI examination was 136 (range 24 to 582days). Accordingly there was enough gadolinium-free-time to detect insoluble gadolinium deposits only.

In conclusion, we could show that no hyperintensities on non-enhanced T1 weighted sequences in the clinical dog patients after 2–3 gadodiamide administrations of 0.1 mmol/kg were detactable and that between 1.5 to 2.5 μg gadolinium/g tissue could be determined in the brain of a research beagle dog 35 months after his last gadodiamide injection. The importance and clinical relevance of gadolinium retention of subvisible contents, the pathomechanism and the potential side effects based on Gd deposition still requires further investigation.

## References

[1] Kanda T, Ishii K, Kawaguchi H, Kitajima K, Takenaka D. High signal intensity in the dentate nucleus and globus pallidus on unenhanced T1-weighted MR images: relationship with increasing cumulative dose of a gadolinium-based contrast material. Radiology. 2014;270(3):834–41. 10.1148/radiol.13131669 . 24475844

[2] Adin ME, Kleinberg L, Vaidya D, Zan E, Mirbagheri S, Yousem DM. Hyperintense Dentate Nuclei on T1-Weighted MRI: Relation to Repeat Gadolinium Administration. AJNR Am J Neuroradiol. 2015;36(10):1859–65. 10.3174/ajnr.A4378 26294649 PMC4878403

[3] Cao Y, Huang DQ, Shih G, Prince MR. Signal Change in the Dentate Nucleus on T1-Weighted MR Images After Multiple Administrations of Gadopentetate Dimeglumine Versus Gadobutrol. AJR Am J Roentgenol. 2016;206(2):414–9. 10.2214/AJR.15.15327 . 26700156

[4] Errante Y, Cirimele V, Mallio CA, Di Lazzaro V, Zobel BB, Quattrocchi CC. Progressive increase of T1 signal intensity of the dentate nucleus on unenhanced magnetic resonance images is associated with cumulative doses of intravenously administered gadodiamide in patients with normal renal function, suggesting dechelation. Invest Radiol. 2014;49(10):685–90. 10.1097/RLI.0000000000000072 . 24872007

[5] Kanda T, Osawa M, Oba H, Toyoda K, Kotoku J, Haruyama T, et al High Signal Intensity in Dentate Nucleus on Unenhanced T1-weighted MR Images: Association with Linear versus Macrocyclic Gadolinium Chelate Administration. Radiology. 2015;275(3):803–9. 10.1148/radiol.14140364 . 25633504

[6] Quattrocchi CC, Mallio CA, Errante Y, Cirimele V, Carideo L, Ax A, et al Gadodiamide and Dentate Nucleus T1 Hyperintensity in Patients With Meningioma Evaluated by Multiple Follow-Up Contrast-Enhanced Magnetic Resonance Examinations With No Systemic Interval Therapy. Invest Radiol. 2015;50(7):470–2. 10.1097/RLI.0000000000000154 . 25756685

[7] Radbruch A, Weberling LD, Kieslich PJ, Eidel O, Burth S, Kickingereder P, et al Gadolinium retention in the dentate nucleus and globus pallidus is dependent on the class of contrast agent. Radiology. 2015;275(3):783–91. 10.1148/radiol.2015150337 . 25848905

[8] Radbruch A, Weberling LD, Kieslich PJ, Hepp J, Kickingereder P, Wick W, et al High-Signal Intensity in the Dentate Nucleus and Globus Pallidus on Unenhanced T1-Weighted Images: Evaluation of the Macrocyclic Gadolinium-Based Contrast Agent Gadobutrol. Invest Radiol. 2015;50(12):805–10. 10.1097/RLI.0000000000000227 . 26523910

[9] Ramalho J, Castillo M, AlObaidy M, Nunes RH, Ramalho M, Dale BM, et al High Signal Intensity in Globus Pallidus and Dentate Nucleus on Unenhanced T1-weighted MR Images: Evaluation of Two Linear Gadolinium-based Contrast Agents. Radiology. 2015;276(3):836–44. 10.1148/radiol.2015150872 . 26079490

[10] Ramalho J, Semelka RC, AlObaidy M, Ramalho M, Nunes RH, Castillo M. Signal intensity change on unenhanced T1-weighted images in dentate nucleus following gadobenate dimeglumine in patients with and without previous multiple administrations of gadodiamide. Eur Radiol. 2016;26(11):4080–8. 10.1007/s00330-016-4269-7 . 26911888

[11] Stojanov DA, Aracki-Trenkic A, Vojinovic S, Benedeto-Stojanov D, Ljubisavljevic S. Increasing signal intensity within the dentate nucleus and globus pallidus on unenhanced T1W magnetic resonance images in patients with relapsing-remitting multiple sclerosis: correlation with cumulative dose of a macrocyclic gadolinium-based contrast agent, gadobutrol. Eur Radiol. 2016;26(3):807–15. 10.1007/s00330-015-3879-9 . 26105022

[12] Tedeschi E, Palma G, Canna A, Cocozza S, Russo C, Borrelli P, et al In vivo dentate nucleus MRI relaxometry correlates with previous administration of Gadolinium-based contrast agents. Eur Radiol. 2016;26(12):4577–84. 10.1007/s00330-016-4245-2 . 26905870

[13] Weberling LD, Kieslich PJ, Kickingereder P, Wick W, Bendszus M, Schlemmer HP, et al Increased Signal Intensity in the Dentate Nucleus on Unenhanced T1-Weighted Images After Gadobenate Dimeglumine Administration. Invest Radiol. 2015;50(11):743–8. 10.1097/RLI.0000000000000206 . 26352749

[14] Hu HH, Pokorney A, Towbin RB, Miller JH. Increased signal intensities in the dentate nucleus and globus pallidus on unenhanced T1-weighted images: evidence in children undergoing multiple gadolinium MRI exams. Pediatr Radiol. 2016;46(11):1590–8. 10.1007/s00247-016-3646-3 . 27282825

[15] Flood TF, Stence NV, Maloney JA, Mirsky DM. Pediatric Brain: Repeated Exposure to Linear Gadolinium-based Contrast Material Is Associated with Increased Signal Intensity at Unenhanced T1-weighted MR Imaging. Radiology. 2017;282(1):222–8. 10.1148/radiol.2016160356 . 27467467

[16] Cao Y, Zhang Y, Shih G, Zhang Y, Bohmart A, Hecht EM, et al Effect of Renal Function on Gadolinium-Related Signal Increases on Unenhanced T1-Weighted Brain Magnetic Resonance Imaging. Invest Radiol. 2016;51(11):677–82. 10.1097/RLI.0000000000000294 . 27272543

[17] Radbruch A, Weberling LD, Kieslich PJ, Hepp J, Kickingereder P, Wick W, et al Intraindividual Analysis of Signal Intensity Changes in the Dentate Nucleus After Consecutive Serial Applications of Linear and Macrocyclic Gadolinium-Based Contrast Agents. Invest Radiol. 2016;51(11):683–90. 10.1097/RLI.0000000000000308 . 27495187

[18] Zhang Y, Cao Y, Shih GL, Hecht EM, Prince MR. Extent of Signal Hyperintensity on Unenhanced T1-weighted Brain MR Images after More than 35 Administrations of Linear Gadolinium-based Contrast Agents. Radiology. 2017;282(2):516–25. 10.1148/radiol.2016152864 . 27513848

[19] Kanda T, Fukusato T, Matsuda M, Toyoda K, Oba H, Kotoku J, et al Gadolinium-based Contrast Agent Accumulates in the Brain Even in Subjects without Severe Renal Dysfunction: Evaluation of Autopsy Brain Specimens with Inductively Coupled Plasma Mass Spectroscopy. Radiology. 2015;276(1):228–32. 10.1148/radiol.2015142690 . 25942417

[20] McDonald RJ, McDonald JS, Kallmes DF, Jentoft ME, Murray DL, Thielen KR, et al Intracranial Gadolinium Deposition after Contrast-enhanced MR Imaging. Radiology. 2015;275(3):772–82. 10.1148/radiol.15150025 . 25742194

[21] Murata N, Gonzalez-Cuyar LF, Murata K, Fligner C, Dills R, Hippe D, et al Macrocyclic and Other Non-Group 1 Gadolinium Contrast Agents Deposit Low Levels of Gadolinium in Brain and Bone Tissue: Preliminary Results From 9 Patients With Normal Renal Function. Invest Radiol. 2016;51(7):447–53. 10.1097/RLI.0000000000000252 . 26863577

[22] Jost G, Lenhard DC, Sieber MA, Lohrke J, Frenzel T, Pietsch H. Signal Increase on Unenhanced T1-Weighted Images in the Rat Brain After Repeated, Extended Doses of Gadolinium-Based Contrast Agents: Comparison of Linear and Macrocyclic Agents. Invest Radiol. 2016;51(2):83–9. 10.1097/RLI.0000000000000242 26606548 PMC4747981

[23] Robert P, Lehericy S, Grand S, Violas X, Fretellier N, Idee JM, et al T1-Weighted Hypersignal in the Deep Cerebellar Nuclei After Repeated Administrations of Gadolinium-Based Contrast Agents in Healthy Rats: Difference Between Linear and Macrocyclic Agents. Invest Radiol. 2015;50(8):473–80. 10.1097/RLI.0000000000000181 26107651 PMC4494686

[24] Robert P, Violas X, Grand S, Lehericy S, Idee JM, Ballet S, et al Linear Gadolinium-Based Contrast Agents Are Associated With Brain Gadolinium Retention in Healthy Rats. Invest Radiol. 2016;51(2):73–82. 10.1097/RLI.0000000000000241 26606549 PMC4747982

[25] Radbruch A, Richter H, Fingerhut S, Martin LF, Xia A, Henze N, et al Gadolinium Deposition in the Brain in a Large Animal Model: Comparison of Linear and Macrocyclic Gadolinium-Based Contrast Agents. Invest Radiol. 2019;54(9):531–6. 10.1097/RLI.0000000000000575 . 31261291

[26] Miller JH, Hu HH, Pokorney A, Cornejo P, Towbin R. MRI Brain Signal Intensity Changes of a Child During the Course of 35 Gadolinium Contrast Examinations. Pediatrics. 2015;136(6):e1637–40. 10.1542/peds.2015-2222 . 26574593

[27] Ramalho J, Ramalho M, AlObaidy M, Nunes RH, Castillo M, Semelka RC. T1 Signal-Intensity Increase in the Dentate Nucleus after Multiple Exposures to Gadodiamide: Intraindividual Comparison between 2 Commonly Used Sequences. AJNR Am J Neuroradiol. 2016;37(8):1427–31. 10.3174/ajnr.A4757 . 27032972 PMC7960266

[28] Roberts DR, Chatterjee AR, Yazdani M, Marebwa B, Brown T, Collins H, et al Pediatric Patients Demonstrate Progressive T1-Weighted Hyperintensity in the Dentate Nucleus following Multiple Doses of Gadolinium-Based Contrast Agent. AJNR Am J Neuroradiol. 2016;37(12):2340–7. 10.3174/ajnr.A4891 27469211 PMC5161565

[29] Tanaka M, Nakahara K, Kinoshita M. Increased Signal Intensity in the Dentate Nucleus of Patients with Multiple Sclerosis in Comparison with Neuromyelitis Optica Spectrum Disorder after Multiple Doses of Gadolinium Contrast. Eur Neurol. 2016;75(3–4):195–8. 10.1159/000445431 . 27054693

[30] Roberts DR, Holden KR. Progressive increase of T1 signal intensity in the dentate nucleus and globus pallidus on unenhanced T1-weighted MR images in the pediatric brain exposed to multiple doses of gadolinium contrast. Brain Dev. 2016;38(3):331–6. 10.1016/j.braindev.2015.08.009 . 26345358

[31] Khant ZA, Hirai T, Kadota Y, Masuda R, Yano T, Azuma M, et al T1 Shortening in the Cerebral Cortex after Multiple Administrations of Gadolinium-based Contrast Agents. Magn Reson Med Sci. 2017;16(1):84–6. 10.2463/mrms.mp.2016-0054 27725576 PMC5600049

[32] Eisele P, Alonso A, Szabo K, Ebert A, Ong M, Schoenberg SO, et al Lack of increased signal intensity in the dentate nucleus after repeated administration of a macrocyclic contrast agent in multiple sclerosis: An observational study. Medicine (Baltimore). 2016;95(39):e4624 10.1097/MD.0000000000004624 27684794 PMC5265887

[33] Schlemm L, Chien C, Bellmann-Strobl J, Dorr J, Wuerfel J, Brandt AU, et al Gadopentetate but not gadobutrol accumulates in the dentate nucleus of multiple sclerosis patients. Mult Scler. 2017;23(7):963–72. 10.1177/1352458516670738 . 27679460

[34] Radbruch A, Haase R, Kieslich PJ, Weberling LD, Kickingereder P, Wick W, et al No Signal Intensity Increase in the Dentate Nucleus on Unenhanced T1-weighted MR Images after More than 20 Serial Injections of Macrocyclic Gadolinium-based Contrast Agents. Radiology. 2017;282(3):699–707. 10.1148/radiol.2016162241 . 27925871

[35] Kuno H, Jara H, Buch K, Qureshi MM, Chapman MN, Sakai O. Global and Regional Brain Assessment with Quantitative MR Imaging in Patients with Prior Exposure to Linear Gadolinium-based Contrast Agents. Radiology. 2017;283(1):195–204. 10.1148/radiol.2016160674 . 27797676

[36] Radbruch A, Haase R, Kickingereder P, Baumer P, Bickelhaupt S, Paech D, et al Pediatric Brain: No Increased Signal Intensity in the Dentate Nucleus on Unenhanced T1-weighted MR Images after Consecutive Exposure to a Macrocyclic Gadolinium-based Contrast Agent. Radiology. 2017;283(3):828–36. 10.1148/radiol.2017162980 . 28273007

[37] Ichikawa S, Motosugi U, Omiya Y, Onishi H. Contrast Agent-Induced High Signal Intensity in Dentate Nucleus on Unenhanced T1-Weighted Images: Comparison of Gadodiamide and Gadoxetic Acid. Invest Radiol. 2017;52(7):389–95. 10.1097/RLI.0000000000000360 . 28195932

[38] Rossi Espagnet MC, Bernardi B, Pasquini L, Figa-Talamanca L, Toma P, Napolitano A. Signal intensity at unenhanced T1-weighted magnetic resonance in the globus pallidus and dentate nucleus after serial administrations of a macrocyclic gadolinium-based contrast agent in children. Pediatr Radiol. 2017;47(10):1345–52. 10.1007/s00247-017-3874-1 . 28526896

[39] Roberts DR, Welsh CA, LeBel DP, 2nd, Davis WC. Distribution map of gadolinium deposition within the cerebellum following GBCA administration. Neurology. 2017;88(12):1206–8. 10.1212/WNL.0000000000003735 . 28202695 PMC11279554

[40] Eisele P, Konstandin S, Szabo K, Ong M, Zollner F, Schad LR, et al Sodium MRI of T1 High Signal Intensity in the Dentate Nucleus due to Gadolinium Deposition in Multiple Sclerosis. J Neuroimaging. 2017;27(4):372–5. 10.1111/jon.12448 . 28569398

[41] Young JR, Orosz I, Franke MA, Kim HJ, Woodworth D, Ellingson BM, et al Gadolinium deposition in the paediatric brain: T1-weighted hyperintensity within the dentate nucleus following repeated gadolinium-based contrast agent administration. Clin Radiol. 2018;73(3):290–5. 10.1016/j.crad.2017.11.005 . 29208312

[42] Moser FG, Watterson CT, Weiss S, Austin M, Mirocha J, Prasad R, et al High Signal Intensity in the Dentate Nucleus and Globus Pallidus on Unenhanced T1-Weighted MR Images: Comparison between Gadobutrol and Linear Gadolinium-Based Contrast Agents. AJNR Am J Neuroradiol. 2018;39(3):421–6. 10.3174/ajnr.A5538 . 29419400 PMC7655328

[43] Quattrocchi CC, Errante Y, Mallio CA, Marinelli L, LoVullo G, Giannotti G, et al Effect of Age on High T1 Signal Intensity of the Dentate Nucleus and Globus Pallidus in a Large Population Exposed to Gadodiamide. Invest Radiol. 2018;53(4):214–22. 10.1097/RLI.0000000000000431 . 29166300

[44] Quattrocchi CC, Ramalho J, van der Molen AJ, Rovira A, Radbruch A, Grec EGREC, et al Standardized assessment of the signal intensity increase on unenhanced T1-weighted images in the brain: the European Gadolinium Retention Evaluation Consortium (GREC) Task Force position statement. Eur Radiol. 2019;29(8):3959–67. 10.1007/s00330-018-5803-6 . 30413951

[45] Rasschaert M, Idee JM, Robert P, Fretellier N, Vives V, Violas X, et al Moderate Renal Failure Accentuates T1 Signal Enhancement in the Deep Cerebellar Nuclei of Gadodiamide-Treated Rats. Invest Radiol. 2017;52(5):255–64. 10.1097/RLI.0000000000000339 28067754 PMC5383202

[46] Jost G, Frenzel T, Lohrke J, Lenhard DC, Naganawa S, Pietsch H. Penetration and distribution of gadolinium-based contrast agents into the cerebrospinal fluid in healthy rats: a potential pathway of entry into the brain tissue. Eur Radiol. 2017;27(7):2877–85. 10.1007/s00330-016-4654-2 27832312 PMC5486780

[47] Gianolio E, Bardini P, Arena F, Stefania R, Di Gregorio E, Iani R, et al Gadolinium Retention in the Rat Brain: Assessment of the Amounts of Insoluble Gadolinium-containing Species and Intact Gadolinium Complexes after Repeated Administration of Gadolinium-based Contrast Agents. Radiology. 2017;285(3):839–49. 10.1148/radiol.2017162857 . 28873047

[48] Lohrke J, Frisk AL, Frenzel T, Schockel L, Rosenbruch M, Jost G, et al Histology and Gadolinium Distribution in the Rodent Brain After the Administration of Cumulative High Doses of Linear and Macrocyclic Gadolinium-Based Contrast Agents. Invest Radiol. 2017;52(6):324–33. 10.1097/RLI.0000000000000344 28323657 PMC5417580

[49] Robert P, Fingerhut S, Factor C, Vives V, Letien J, Sperling M, et al One-year Retention of Gadolinium in the Brain: Comparison of Gadodiamide and Gadoterate Meglumine in a Rodent Model. Radiology. 2018;288(2):424–33. 10.1148/radiol.2018172746 . 29786486

[50] Feldmann J, Kindness A, Ek P. Laser ablation of soft tissue using a cryogenically cooled ablation cell. J Anal Atom Spectrom. 2002;17(8):813–8. 10.1039/b201960d WOS:000177254600010.

[51] Bishop DP, Clases D, Fryer F, Williams E, Wilkins S, Hare DJ, et al Elemental bio-imaging using laser ablation-triple quadrupole-ICP-MS. J Anal Atom Spectrom. 2016;31(1):197–202. 10.1039/c5ja00293a WOS:000367315200015.

[52] Fingerhut S, Niehoff AC, Sperling M, Jeibmann A, Paulus W, Niederstadt T, et al Spatially resolved quantification of gadolinium deposited in the brain of a patient treated with gadolinium-based contrast agents. J Trace Elem Med Biol. 2018;45:125–30. 10.1016/j.jtemb.2017.10.004 . 29173468

[53] Boyken J, Frenzel T, Lohrke J, Jost G, Pietsch H. Gadolinium Accumulation in the Deep Cerebellar Nuclei and Globus Pallidus After Exposure to Linear but Not Macrocyclic Gadolinium-Based Contrast Agents in a Retrospective Pig Study With High Similarity to Clinical Conditions. Invest Radiol. 2018;53(5):278–85. 10.1097/RLI.0000000000000440 29319556 PMC5902136

[54] Frenzel T, Apte C, Jost G, Schockel L, Lohrke J, Pietsch H. Quantification and Assessment of the Chemical Form of Residual Gadolinium in the Brain After Repeated Administration of Gadolinium-Based Contrast Agents: Comparative Study in Rats. Invest Radiol. 2017;52(7):396–404. 10.1097/RLI.0000000000000352 28125438 PMC5464750

[55] Kartamihardja AA, Nakajima T, Kameo S, Koyama H, Tsushima Y. Distribution and clearance of retained gadolinium in the brain: differences between linear and macrocyclic gadolinium based contrast agents in a mouse model. Br J Radiol. 2016;89(1066):20160509 10.1259/bjr.20160509 27459250 PMC5124816

[56] Quattrocchi CC, Mallio CA, Errante Y, Beomonte Zobel B. High T1 Signal Intensity in Dentate Nucleus after Multiple Injections of Linear Gadolinium Chelates. Radiology. 2015;276(2):616–7. 10.1148/radiol.2015150464 . 26203714

[57] Korkusuz H, Ulbrich K, Welzel K, Koeberle V, Watcharin W, Bahr U, et al Transferrin-coated gadolinium nanoparticles as MRI contrast agent. Mol Imaging Biol. 2013;15(2):148–54. 10.1007/s11307-012-0579-6 . 22811020

[58] El-Khatib AH, Radbruch H, Trog S, Neumann B, Paul F, Koch A, et al Gadolinium in human brain sections and colocalization with other elements. Neurol Neuroimmunol Neuroinflamm. 2019;6(1):e515 10.1212/NXI.0000000000000515 30568993 PMC6278849

[59] Kiviniemi A, Gardberg M, Ek P, Frantzen J, Bobacka J, Minn H. Gadolinium retention in gliomas and adjacent normal brain tissue: association with tumor contrast enhancement and linear/macrocyclic agents. Neuroradiology. 2019;61(5):535–44. 10.1007/s00234-019-02172-6 . 30710184

[60] Wang S, Hesse B, Roman M, Stier D, Castillo-Michel H, Cotte M, et al Increased Retention of Gadolinium in the Inflamed Brain After Repeated Administration of Gadopentetate Dimeglumine: A Proof-of-Concept Study in Mice Combining ICP-MS and Micro- and Nano-SR-XRF. Invest Radiol. 2019;54(10):617–26. 10.1097/RLI.0000000000000571 . 31033673

[61] Varatharaj A, Galea I. The blood-brain barrier in systemic inflammation. Brain Behav Immun. 2017;60:1–12. 10.1016/j.bbi.2016.03.010 . 26995317

